# Pregnancy in a Young Patient with Metastatic HER2-Positive Breast Cancer—Between Fear of Recurrence and Desire to Procreate

**DOI:** 10.3390/curroncol30050364

**Published:** 2023-05-08

**Authors:** Cristina Marinela Oprean, Andrei Dorin Ciocoiu, Nusa Alina Segarceanu, Diana Moldoveanu, Alexandra Stan, Teodora Hoinoiu, Ioana Chiorean-Cojocaru, Daciana Grujic, Adelina Stefanut, Daniel Pit, Alis Dema

**Affiliations:** 1 ANAPATMOL Research Center, ‘Victor Babes’ University of Medicine and Pharmacy of Timisoara, 300041 Timisoara, Romania; 2 Department of Oncology, ONCOHELP Hospital Timisoara, Ciprian Porumbescu Street, No. 59, 300239 Timisoara, Romania; 3 Department of Oncology, ONCOMED Outpatient Unit, Ciprian Porumbescu Street, No. 59, 300239 Timisoara, Romania; 4 Department of Oncology, City Clinical Emergency Hospital of Timisoara, Victor Babes Blvd. No. 22, 300595 Timisoara, Romania; 5 Department of Clinical Practical Skills, “Victor Babes” University of Medicine and Pharmacy, Eftimie Murgu Sq. Nr. 2, 300041 Timisoara, Romania; 6 Center for Advanced Research in Cardiovascular Pathology and Hemostaseology, “Victor Babes” University of Medicine and Pharmacy, 300041 Timisoara, Romania; 7 Department of Obstetrics and Gynaecology, Faculty of Medicine and Pharmacy, “Victor Babes” University of Medicine and Pharmacy, 300041 Timisoara, Romania; 8 Department of Plastic and Reconstructive Surgery, “Victor Babes” University of Medicine and Pharmacy, Eftimie Murgu Sq. Nr. 2, 300041 Timisoara, Romania; 9 Department of Psichology & Sociology, West University, Timisora, Blvd. No. 4, Vasile Pârvan, 300223 Timisoara, Romania

**Keywords:** metastatic breast cancer, pregnancy, trastuzumab, chemotherapy, pregnancy-associated breast cancer, PABC

## Abstract

Breast cancer is the most frequent neoplasm among women and the second leading cause of death by cancer. It is the most frequent cancer diagnosed during pregnancy. Pregnancy-associated breast cancer is defined as breast cancer that is diagnosed during pregnancy and/or in the postpartum period. Data about young women with metastatic HER2-positive cancer who desire a pregnancy are scarce. The medical attitude in these clinical situations is difficult and nonstandardized. We present the case of a 31-year-old premenopausal woman diagnosed in December 2016 with a stage IV Luminal HER2-positive metastatic breast cancer (pT2 N0 M1 hep). The patient was initially treated by surgery in a conservative manner. Postoperatively, the presence of liver metastases was found by CT investigation. Consequently, line I treatment (docetaxel l75 mg/m² iv; trastuzumab 600 mg/5 mL sq) and ovarian drug suppression (Goserelin 3.6 mg sq at 28 days) was administered. After nine cycles of treatment, the patient’s liver metastases had a partial response to the therapy. Despite having a favorable disease evolution and a strong desire to procreate, the patient vehemently refused to continue any oncological treatment. The psychiatric consult highlighted an anxious and depressive reaction for which individual and couple psychotherapy sessions were recommended. After 10 months from the interruption of the oncological treatment, the patient appeared with an evolving pregnancy of 15 weeks. An abdominal ultrasound revealed the presence of multiple liver metastases. Knowing all the possible effects, the patient consciously decided to postpone the proposed second-line treatment. In August 2018, the patient was admitted in the emergency department with malaise, diffuse abdominal pain and hepatic failure. Abdominal ultrasound found a 21-week-old pregnancy which had stopped in evolution, multiple liver metastases and ascites in large quantity. She was transferred to the ICU department where she perished just a few hours later. Conclusions/Discussion: From a psychological standpoint, the patient had an emotional hardship to make the transition from the status of a healthy person to the status of a sick person. Consequently, she entered a process of emotional protection of the positive cognitive distortion type, which favored the decision to abandon treatment and try to complete the pregnancy to the detriment of her own survival. The patient delayed the initiation of oncological treatment in pregnancy until it was too late. The consequence of this delay in treatment led to the death of the mother and fetus. A multidisciplinary team worked to provide this patient with the best medical care and psychological assistance throughout the course of the disease.

## 1. Introduction

Breast cancer is the most common malignancy amongst women. It is the second leading cause of death by cancer [1]. In the US, approximately 11% of 230,000 new cases of invasive breast cancer are diagnosed in women under the age of 45 [2]. In recent years, there has been an increase in the incidence of breast cancer in women under 40 [3]. In the last five decades, since the 1970s, there has been a trend in postponing pregnancies to a more mature age (delaying childbearing) [4]. As the incidence of breast cancer increases with age, more and more women are diagnosed with cancer during pregnancy, and on the other hand, despite being diagnosed with cancer, some women want a child at any cost [5]. Pregnancy-associated breast cancer (PABC) is defined as breast cancer that is diagnosed during pregnancy and/or the postpartum period (Shao 2020). Breast cancer is the most common malignancy diagnosed during pregnancy, with an increase in incidence in recent decades [6]. The incidence of breast cancer in pregnancy is estimated at 1/3000 pregnancies, about 3% of all breast cancers [7,8,9]. Pregnancy that occurs before or concurrently with a diagnosis of breast cancer is more likely to result in death and decreased disease-free survival [10]. Breast cancer during pregnancy should be treated as much as possible following the recommendations of the guidelines for breast cancer in nonpregnant women. Chemotherapy is contraindicated in the first trimester of pregnancy due to an increased risk of induction of fetal malformation, as it is the period in which oncogenesis is formed [6]. The most common fetal malformations are deafness, gonadal malformations, cardiac complications (arrhythmias, ischemia and thrombocytopenia), or cognitive disabilities [11]. Moreover, administering chemotherapy in the first trimester of the pregnancy carries a 17% risk of inducing a miscarriage [12]. Chemotherapy can be safely administered during the second and third trimesters of pregnancy [5,13]. The use of chemotherapy during the second and third trimesters of pregnancy may be associated in approximately 10–20% of cases with an increase in the number of obstetric and fetal complications, including hypertensive disorders, restriction of intrauterine growth and premature birth [13]. Anthracycline-based chemotherapy is the standard therapy and can be safely administered to pregnant breast cancer patients during the second and third trimesters of pregnancy. Clinical experience in the use of taxanes in this subtype of patient is limited [13,14]. Endocrine therapy as well as anti-HER2 therapy (the monoclonal antibody trastuzumab) should be avoided during pregnancy, and treatment with these agents should be postponed until after birth [6]. Anti-HER2 therapy is associated with an increased risk of anhydramnios (absence of amniotic fluid leading to fetal lung hypoplasia and postpartum respiratory distress or even intrauterine death) [15,16,17].

### Psychological Implications of Breast Cancer Diagnosis

The diagnosis of breast cancer has implications not only on a physical level but also on a psychological level that are closely related to the disease itself but also to the specifics of the treatments applied. Thus, although the efficacy of these treatments has increased over time, one third of patients do not follow an adjuvant treatment, because they refuse it [18]. Understanding the psychological reality of these patients as well as the factors that contribute to refusing or stopping treatment is particularly important.

They may have a reaction of distrust or of denial; they may face a feeling of uncertainty. During this transition period, patients face multiple stressors, and the lack of similar experiences in their own life or in the life of those in their families makes the coping process difficult [19].

Regarding treatment compliance, among the factors that can influence it, we include the existence of depression, the level of medical knowledge, beliefs about treatment and the trust in the medical system [20]. In the case of young women diagnosed with breast cancer, the psychological problem presented above is frequently added to the desire to have children. In general, women’s desire to have children can be emotionally grounded or may be the result of society’s expectations; it may be based on the desire to please their life partner [21], or it may mean the end of loneliness because the mother has a person who can love her [22]. In addition, the diagnosis of breast cancer can intensify this desire and give it a new symbolic meaning, such as being normal and able to achieve something beautiful, as opposed to death [23]. In the context of breast cancer, young women face a strong sense of injustice derived from the fear of not being able to give birth and the need to change their future plans regarding having a child, which can affect the achievement of life goals [24].

It is also worth noting that young women newly diagnosed with breast cancer report that their most important concerns are children and family, even at the expense of their own survival [25]. A study involving breast cancer survivors and their partners shows that the experience of the disease did not diminish the motivation to have a biological child for either women or their husbands [23]. 

Data about young women with metastatic HER2-positive cancer who desire a pregnancy are scarce. The medical conduct in these clinical situations is difficult and nonstandardized. Patients need strong emotional support to help them make a decision about procreation and fear of recurrence. To the best of our knowledge, this is the first case report about an unfavorable outcome of a pregnant mother with metastatic HER2-positive breast cancer.

## 2. Case Presentation

**Figure 1:**
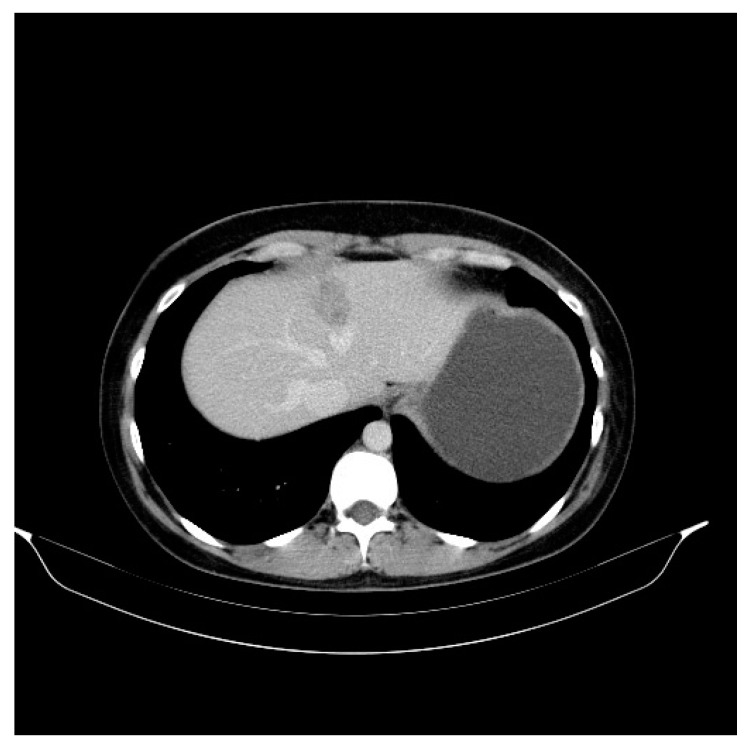
Liver metastasis in 31-year-old women with infiltrative ductal breast carcinoma (IDC), G2, with an “in situ” ductal component of the comedo type. Axial noncontrast abdominal CT image performed in 2016 shows a liver metastasis (31 mm diameter) with an ill-defined area of low attenuation and faint high attenuation.

**Figure 2:**
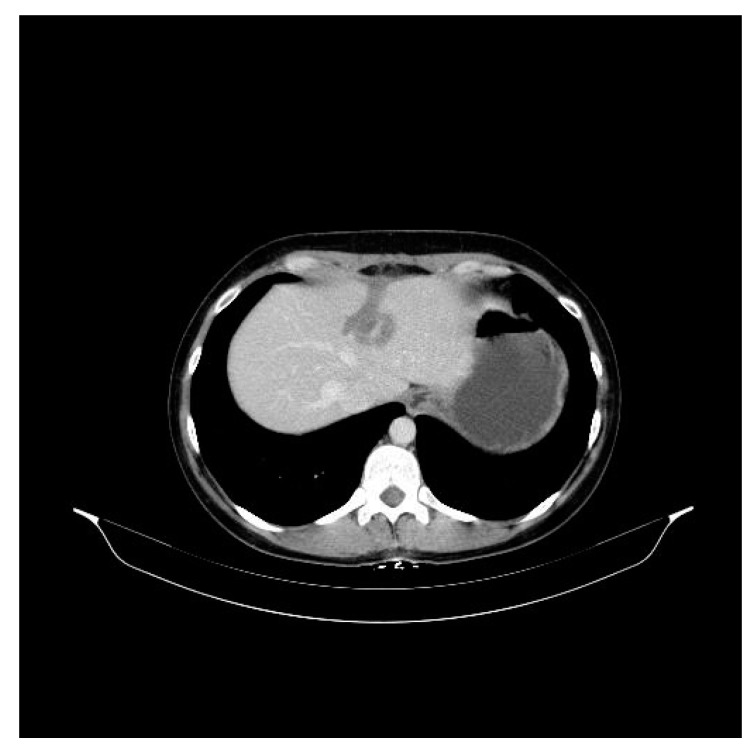
Aspect on the abdominal CT in 2017 after medication with docetaxel 75 mg/m^2^ iv, trastuzumab 600 mg/5 mL sq./21 days and medical ovarian suppression (goserelin 3.6 mg sq./28 days) with the liver metastasis (15 mm diameter).

**Figure 3:**
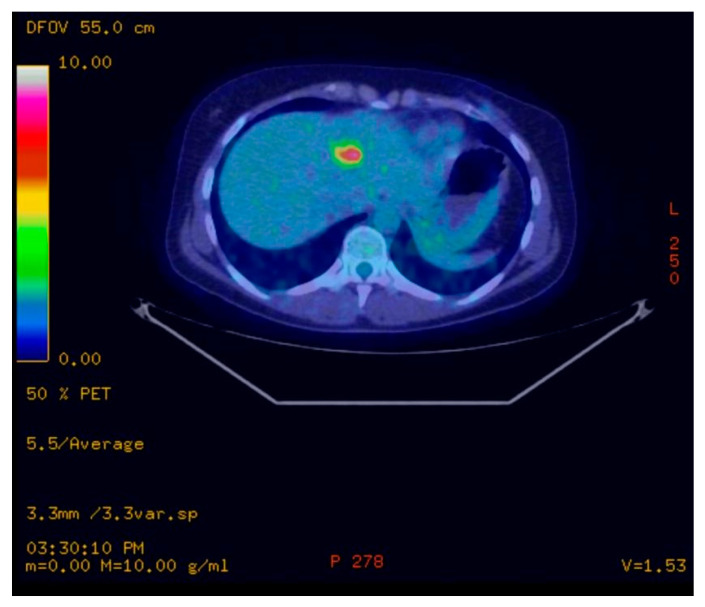
PET/CT scans performed in 2017 showing a single liver lesion of 22.3 mm in the 2nd segment with intense metabolic activity (SUV = 6.4) described as a single liver metastasis.

**Figure 4:**
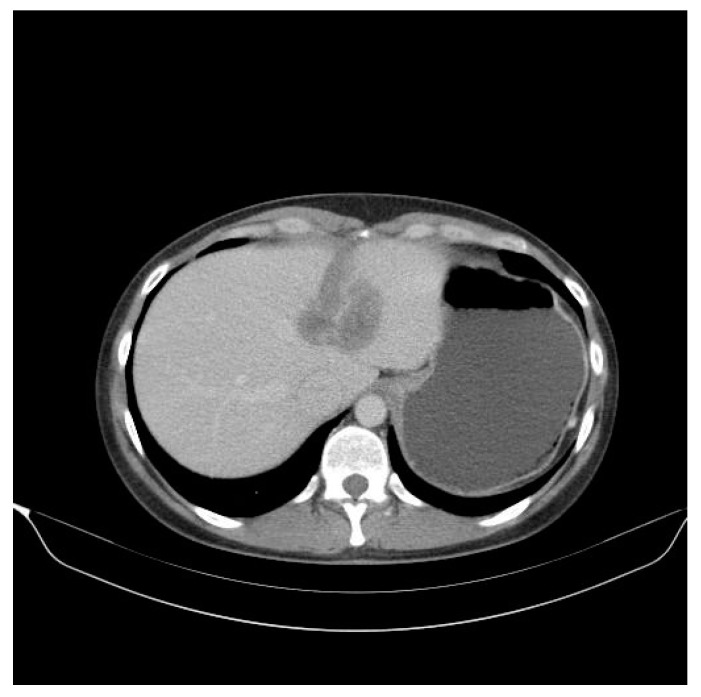
Follow-up abdominal CT performed in 2018 at 6 months after two ablation sessions of the liver metastasis with an MW of 6 and 4 min at 32 W with a temperature set at 96 degrees highlights a progressive disease of the liver metastases at 55 mm diameter.

We present the case of a premenopausal, employed, unmarried, urbanite, nulliparous, 31-year-old patient with a negative personal and familial medical history. In December 2015, she was diagnosed clinically and by imaging with a tumor formation in her left breast, in the lower inner quadrant of 25/13/16 mm. The patient delayed the definite diagnosis by performing the biopsy 1 year later, in November 2016. The pathology examination revealed a hormone-dependent (ER = 90, PR = 30%), Ki67 = 30%, HER2 = 3 + (positive) infiltrative ductal breast carcinoma (IDC), G2, with an “in situ” ductal component of the comedo type. The preoperative assessment consisted of performing a bilateral mammogram and a bilateral breast MRI, thus avoiding the diagnosis of liver metastases at the time of diagnosis. The patient was initially clinically staged T2N0, being considered a nonmetastatic disease. On 8 December 2016, a left breast conserving surgery and a sentinel node biopsy was performed. Postoperative histopathological examination confirmed a infiltrative ductal carcinoma with components of “in situ” breast carcinoma; Nsn = 0/2 lymph nodes examined (pT2Nsn0). Immunohistochemistry (IHC) revealed an RE = 80%, RP = 20%, Ki 67 = 30% and HER2 = 3 + (positive) profile. On the CT scan performed during the pretherapeutic assessment on 19 December 2016, two liver metastases of 31 mm and 12 mm were observed in the 4th and 2nd segment (Figure 1). The CA15-3 tumor marker had a value of 86.8 U/mL. First-line treatment with docetaxel 75 mg/m^2^ iv, trastuzumab 600 mg/5 mL sq./21 days and medical ovarian suppression (goserelin 3.6 mg sq./28 days) was initiated. The treatment was well tolerated by the patient, with no significant side effects. The CT scan performed in May 2017, after six cycles of treatment, highlighted a partial response: the liver lesion in the 4th segment had decreased dimensions (15 mm vs. 31 mm), and the lesion in segment II was in complete remission (Figure 2). The treatment was continued for three more cycles. The PET-CT performed in July 2017 described a single liver lesion of 22.3 mm in the 2nd segment, intensely metabolically active (SUV = 6.4) (Figure 3). Transaminases, both after the 6th and 9th cycle, were within normal limits. For the remaining liver injury, the patient performed, in August 2017, two ablation sessions with an MW of 6 and 4 min at 32 W with a temperature set at 96 degrees. Due to the favorable evolution of the disease and the strong desire to have a child, the patient refused to continue any oncological treatment, including ovarian ablation and the recommended hormone therapy (tamoxifen). The psychiatric consultation highlighted an anxious and depressive reaction for which individual and couple psychotherapy sessions were recommended. The patient only attended regular individual psychotherapy sessions, because at that time she had no life partner. Three months later, in January 2018, the CT scan highlighted a progressive disease of the 4th liver segment lesion (from 33 mm to 55 mm) (Figure 4). She was proposed a 2nd-line treatment with the resumption of the ovarian suppression with goserelin, but the patient refused any additional oncological treatment. In July 2018, the patient presented to the clinic after an emergency consultation, being pregnant for 15 weeks and complaining of abdominal pain. Ultrasound revealed multiple hepatic lesions suggestive of metastases. Tumor marker CA 15-3 had a value of 2400 U/mL, and the common liver tests were slightly above the normal limit (AST = 50 U/L; ALAT = 80 U/L, TB = 1.8 mg/dL). Although the patient was informed about the prognosis at this stage of the disease and about the potential vital risks in case of pregnancy, she wanted to keep the pregnancy, being aware of the consequences of this decision on the evolution of the disease and survival. She did not consult with her parents or with her life partner. Psychological counselling was carried out individually only with the patient, who did not want the family present at the counselling sessions. The psychiatric evaluation performed did not find the existence of any psychiatric pathology that would affect her decision making. We proposed the initiation of chemotherapy, but the patient refused, wanting to go abroad for a second-opinion consultation. She hoped that this consultation would give her a lifesaving solution for the clinical situation she was in. At the end of August 2018, the patient was transported to the emergency department with an affected general state, diffuse abdominal pain and liver failure (AST = 6017 U/L, ALT = 782 U/L; TB = 6.67 mg/dL). The abdominal ultrasound that was performed in the OBGYN department revealed a 21-week-old pregnancy halted in evolution, the absence of fetal heartbeat, biometrics corresponding to an 18-week-old pregnancy, amniotic fluid in normal quantity, multiple liver metastases that almost entirely occupied the liver parenchyma and ascites in large quantities. Given the serious general condition and the impossibility of evacuating the pregnancy by surgery (uterine curettage or total hysterectomy) due to the very high risk of anesthetic death, she was transferred to the intensive care unit where she died a few hours later. 

The psychological aspects involved in this case are, first of all, the postponement by the patient of the investigations that could specify the diagnosis with certainty. This postponement was justified by the patient on the basis of the belief that it could not be a serious illness considering her young age. With the initiation and then continuation of chemotherapy, its side effects also appeared. Although the patient’s body tolerated the medication well, the psychosocial impact of the disease and treatment was considerable. The patient was a very active person and the social and professional limitations resulting from her illness changed not only her life routine but also reduced her access to the situations that could create meaning and significance. These limitations also contributed to the reduction in social support. The patient was the only child in the family, so she thought it was her duty to protect her parents from the negative psychological impact of finding out the diagnosis. Therefore, she presented the situation to her parents in a much better light than it was in reality, and when the treatment was stopped, they thought that it was no longer necessary to continue it. This state of affairs had the consequence of depriving the patient from adequate support from her parents. 

The patient expressed positive beliefs about herself and about her ability to overcome the disease. She also expressed an optimistic view of the course of the disease, believing that it could be stopped even if she interrupted treatment—this view being in fact a way of denying the severity of the disease. Even though she generally acknowledged the severity of the disease, she could not accept that she herself could be in a serious situation.

Although physically the treatment was well tolerated by the patient, she stated that the treatment “does her no good” and that it “changes” her. The way she perceived herself had changed, and she was not satisfied with this new situation. She did not complain about the bodily changes that occurred, but she pointed out the fact that before she was a cheerful, active, full-of-life person, and now she felt deprived of energy and permanently tired. In an attempt to return to her previous condition, once the treatment was stopped, she resumed her professional life, although she could have benefited from another 8 months of medical leave. Involvement in a relationship and then the onset of pregnancy are part of the same attempt to return to her previous life when she was a healthy person.

## 3. Discussion

Breast cancer, malignant melanoma, cervical cancer, lymphoma and acute leukemia are the most common cancers diagnosed in pregnancy. The occurrence of pregnancy in a patient with metastatic or recurrent disease is a rare and difficult clinical situation. There are few publications in the literature that present these clinical situations. The vast majority of published cases present clinical situations in which the evolution was favorable and the pregnancy was completed. Mauricio Burotto presents such a case in a patient with metastatic malignant melanoma [26]. Clinical cases with successful outcome have also been published: pregnancy in a patient with recurrent ovarian angiosarcoma [27], pregnancy in a patient with recurrent and high-grade metastatic osteosarcoma [28]. Isolated cases have been published in the literature on pregnant patients treated with trastuzumab. Thus, Michelle A. Fanare described a case of HER2-positive metastatic breast cancer in a 27-week-pregnant woman treated with trastuzumab and vinorelbine weekly. Although she suffered an anhydramnios as a side effect, the patient managed to give birth to a healthy baby [16]. One of our explanations would be that since this patient was 27 weeks pregnant, the exposure to trastuzumab during pregnancy was not long-lasting. In our patient, anti-HER2 therapy may not have been a treatment option, requiring longer-term exposure to trastuzumab and probably with more severe side effects. Most likely, our treatment option would have been the use of anthracyclines or taxanes (especially considering the good response to taxanes we had at the first administration), while the use of trastuzumab would have been postponed until after birth.

Pregnancy associated with breast cancer is defined differently in different clinical trials, and this difference explains the inhomogeneous results related to the prognosis of these patients. A recent meta-analysis of 54 articles with 76 included clinical trials that analyzed the prognosis of patients with pregnancy associated with breast cancer concluded that this clinical situation is associated with poor prognosis. However, whether PABC has a worse prognostic is controversial [29]. A meta-analysis published in 2016 showed an increased risk of death in women with PABC compared with non-PABC [10]. Other recent studies found no significant difference in the prognostic of PABC compared with women with non-PABC [30,31,32,33]. Case-control studies found that the prognosis is more unfavorable in breast cancer in pregnancy, but when analyzing the data on the TNM stage of the disease, it was observed that the prognosis is not significantly different [34]. For patients who received chemotherapy during pregnancy, the survival data were comparable to those of nonpregnant patients [35,36]. Most children exposed to chemotherapy during intrauterine life have no significant complications [37].

In the case of our patient, the initiation of oncological treatment during pregnancy was postponed until it was too late, which required a multidisciplinary collaboration between the oncologist and obstetrician for the relative benefits of the fetus. Clinical trials have shown that chemotherapy can be safely administered in the second and third trimesters, starting at week 16 of pregnancy. In the selection of treatment, the criteria involve the time of diagnosis, hormonal status and trimester of pregnancy [12]. One of the open questions would be “if we had started chemotherapy, would the patient have managed to complete the pregnancy?” Among the negative prognostic factors present in this case, we mention pregnancy, stage of the disease (patient with metastatic disease at onset), progressive disease at the time of pregnancy, young age and overexpression of HER2. We can assume that if we had initiated chemotherapy, there would have been a chance that the patient would have completed the pregnancy. Unfortunately, the consequence of the delay of the oncological treatment resulted in the death of the fetus and the mother.

The cause of fetal death in utero may be due to hypoxia due to placental detachment with disseminated intravascular coagulation [38] or placental metastases that may affect the fetal circulation if they exceed the villous space of the placenta or may not affect the fetus if they remain at that level [39]. Other causes can be intrauterine growth restrictions or even fetal malformation. Placental metastases are rarely described in the literature, occurring in approximately 17% of cases (4 cases out of 24) [40].

If we refer to the psychological implications associated with this case, we note that finding a diagnosis of a potentially fatal disease can greatly change a person’s assumptions about the world and about themself. Reconstructing the worldview requires both emotional and cognitive processing, at a time when the enormity of the threat and emotions can be overwhelming [41]. Young patients newly diagnosed with breast cancer report this passage through various emotional states, which they describe as an “emotional rollercoaster” [19]. From a cognitive perspective, people can resort to three different processes to keep negative emotions aside: cognitive avoidance, emotional avoidance and cognitive distortion. Cognitive avoidance refers to concentrating attention voluntarily or automatically elsewhere to avoid thoughts or images that create distress. Emotional avoidance is a dissociative mechanism by which the person is able to talk about stressful, serious events without having emotional reactions. The last type of avoidance—cognitive distortion—refers to the tendency to operate within a positive bias expressed, for example, by overestimating the probability of experiencing positive events [42].

In the case presented in this study, the hypothesis is that in trying to protect herself emotionally from the implications of the severe diagnosis she was facing, the patient resorted to cognitive distortion. The patient’s beliefs about herself before the disease outlined the image of a strong, active person with multiple resources, able to face adversity. These beliefs, which are the basis of an optimistic vision, and which support a fighting spirit against the disease, can make an easy and imperceptible transition to an optimism that is not objectively sustained. This transition was probably made when she gave up treatment, considering the personal resources available to her ensured her success in dealing with the disease. It is also possible that this transition was triggered by the news of encouraging treatment results, known to be the mechanism involved in cognitive distortion through which a person filters a certain category of information (in this case, the negative ones) and focuses on other categories of information (in this case positive). However, the consequence of excluding negative information can be extremely harmful, because it leads to a reduction in the perception of the threat and to the endangerment of the person. Another aspect that can be observed in this case is the fact that the extremely positive assumptions about herself that existed before the diagnosis was made were not changed after the diagnosis or during the treatment. The discrepancy between self-image and the reality of the disease, in which there were aspects that were out of her personal control, created an emotional discomfort expressed by the patient. However, this discomfort was solved not by updating her self-image by integrating the fact that there are situations that are not under one’s control but by the positive cognitive distortion of reality as we described previously. An explanatory hypothesis for this way of resolving the internal conflict may be that the acceptance of the personal lack of control in the context of the disease would have led to an intensified perception of the disease threat at a level that the patient would not have been able to cope with. This loss of control and the feeling of being trapped in a system that dictates what to do is reported in the literature by young patients diagnosed with breast cancer [19].

In the present case, the protective attitude that the patient adopted towards the family also draws attention. Although the family was informed of the diagnosis, the situation presented was better than the real one. This position may also be based on the positive beliefs about oneself presented above and is also observed in other young patients with breast cancer [19].

Even in the case of women with early stage breast cancer, the usual medical recommendation is to wait at least two years after the end of the treatment before becoming pregnant, due to the fact that most recurrences occur during this time [42]; when the pregnancy occurred, our patient decided to try to complete it. Despite the risks to her own health, she made the decision, saying that she “wants to leave something behind”, which suggests the possible motivation for a symbolic immortality, as evidenced in the literature [23].

## 4. Conclusions

Pregnancy generally appears to be safe for fetuses, newborns and mothers, without requiring the need for some long-term clinical trials to provide more and more reliable information to doctors and patients [43]. We are, in fact, faced with an enormous challenge. Pregnancy after cancer is an area still being explored and is a fascinating stimulus of knowledge to dedicate oneself to oncology and women’s health.

Our conclusion is that in the present case, an important role from a psychological point of view was played by the patient’s difficulty to make the transition from the status of a healthy person to the status of a sick person, as well as not updating her self-image according to the new context. This had, as a consequence, an entry into emotional protection processes, such as positive cognitive distortion, which favored the decision to abandon the treatment and the attempt to complete the pregnancy at the expense of her own survival. The patient delayed the initiation of oncological treatment in pregnancy until it was too late. The consequence of this delay in treatment led to the death of the mother and fetus. A multidisciplinary team was committed to providing this patient with the best medical care and psychological assistance throughout the course of the disease. Maybe a strong family and social support, together with more intensive psychological intervention, would have made the evolution of this case different.

## Data Availability

The data presented in this study are available on request from the corresponding author. The data are not publicly available.
